# The computational analyses, molecular dynamics of fatty-acid transport mechanism to the CD36 receptor

**DOI:** 10.1038/s41598-021-01373-5

**Published:** 2021-12-01

**Authors:** Jihane Akachar, Catherine Etchebest, Rachid El Jaoudi, Azeddine Ibrahimi

**Affiliations:** 1 Laboratory of Biotechnology (MedBiotech), Rabat Medical and Pharmacy School, Mohammed V University in Rabat, Rabat, Morocco; 2 UMR_S 1134, INSERM, DSIMB, Université Paris Diderot, Sorbonne Paris Cite, Paris, France

**Keywords:** Biochemistry, Biophysics, Computational biology and bioinformatics, Drug discovery, Structural biology, Systems biology

## Abstract

The transmembrane glycoprotein CD36, which is responsible of the metabolic disorders, and the elevated intake of fat induces lipid buildup, is a multifunctional scavenger receptor signaling those functions in high-affinity tissue uptake of long-chain fatty acids. In this study, we used series of molecular dynamics simulations of the wild type and mutants types K164A CD36 protein interacting with one palmitic acid (PLM) besides simulations of the wild type interacting with the three PLM to find out the mechanism of the functioning of the complex CD36/Fatty acids and the unraveling of the role of the mutation. Additionally we determined whether Lys164, mostly exposed to protein surface, played important roles in fatty acid uptake. These simulations revealed, the conformational changes induced by Lys164 residue and the altered interactions induced by the mutagenesis of surface lysine that was badly influencing the folding, utility, solubility, and stability form of the variant. Furthermore, Lys164 residue provided the structural basis of forming an opening at the region of principal portal for the dissociation of palmitic acid. The results of our simulations revealed hole two fatty acids found in CD36 cavity structure and it was the most preferred to CD36 structure stabilization.

## Introduction

CD36 is a multifunctional transmembrane and glycoprotein member of the class B scavenger receptor family^1^. CD36 binds many ligands including long chain fatty acids, collagen, hexarelin, thrombospondin, and erythrocytes infected with Plasmodium falciparum^2,3^. It oxidized low density lipoprotein (oxLDL), native lipoproteins, and phospholipids^4^. CD36 plays a significant function in cell signaling capabilities, in fatty acid uptake, angiogenesis, phagocytosis and it is also implicated in a list of pathways related to cellular resistance, inflammation, and the evolution of atherosclerosis^5–7^. Recent research has shown that CD36 is involved in the regulation of calcium levels, phospholipase activation, and eicosanoid generation^8–11^. CD36 variants had been associated to blood lipid levels alterations as well to susceptibility to the metabolic modifications^12,13^.

Information associated to the transport function of CD36 is still incomplete; yet, many advanced studies have lately expressed that it includes two transmembrane helices, which are inter and lay in a ~ 47 kDa exoplasmic region^14^. CD36 contains an exoplasmic part which is a range of hydrophobic 184–204 amino acids that could work with the cell membrane^15^. The fixation region of CD36 for fatty acids, the LDL and hexarelin have been placed to residues (155–183)^16^ while, for thrombospondin-1 research has displayed to be 93–120 amino acids^17^. The structure of LIMP-2, a part of the CD36 super family, has appeared as a considerable hole that goes by through the whole extent of the protein^18^. This cavity should be considered the site through which fatty acids go over as they diffuse across the cell membrane. The CD36: CIDRα 2.8 complex, which was subjected to crystallization trials at pH 8.0, arranges an open conformation. A cutting through a surface illustration of CD36 displayed the main core cavity was identified only by two palmitic acids^14^. A significant properties in CD36 that are (oxLDL) and oxidized phosphatidylcholine engage in a common fixation region of the Fatty acid, as these two molecules, were suggested to interact with Lys-164 and Lys-166^19^. In addition, fatty acids can be cross-linked to K164 of CD36 and are then thought to move to penetrate the central CD36 cavity and pass through the CD36 exoplasmic part to the membrane surface. Also, the cysteine-rich interdomain region α (CIDRα) region bonding inter Lys164 and the nonpolar hole, block the gate of the fatty acid. CD36 functions in a fatty acid absorption and transmission signal can be blocked by sulfo-*N*-succinimidyloleate, which link lysine 164 within a nonpolar hole shared by several CD36 molecules, e.g. fatty acid and oxLDL20^20,21^. CD36 also mediated FA uptake and signaling in CHO cells expressing mutant CD36 (K164), this can be related to that short-chain-alkyl of FA mediated the FA in relative orientation and position^22,23^. The second that it is in the surface of the pocket, the carboxyl group of the FA can compose electrostatic interactions with Lys-164, and this pathway followed by CD36 conformational deviation to activate FA uptake and/or signaling. This analysis is based on the observation of the mutated CD36 expression, where the substitution of even one residue Lys to Ala, triggers a signaling cascade where its stimulation by FA binding. Thus, further, Lys-164 residues mediating as well as the interaction of FA mechanism with CD36 are still unknown to the present time. As fatty acid uptake is linked to fatty acid metabolism and atherosclerosis, it would be interesting to see how we can prevent fatty acid uptake as a proof of principle of targeting this region of CD36. Although that the prevalence of studies that CD36 is an essential receptor is involved in the fat metabolism, detection, signaling pathways in specific cells. It also, could imagine that a potential drug target for the treatment of diabetes, atherosclerosis and obesity and their features of cardiovascular diseases. Therefore, it is of a great significance to identify the transport mechanism of CD36.

In this paper, we attempted to study the complex structures of wild-type and mutated CD36/Fatty acid (FA) by molecular dynamic approaches, with the aim of providing detailed mechanism for the effect of Mutagenese Lys164 in fatty acid transport and rate. Thus, the determination of mutated structure is important as no structural information is available in the protein structure databases. Firstly, we used a computational approach to construct a three-dimensional structural model then, we utilized molecular dynamic to identify fatty acid binding site, transport trajectory and rate. Based on these computational studies, the structural analysis of Lys164 mutagenesis with FA interaction in the binding protein pocket has been documented and exposed to influence FA transfer. Yet, the findings could provide a framework for how lys164 might induce dysfunction of CD36-regulated FA which can be considered one of the parameters in protein stability. This could be a target for discovering a new drug by finding new mechanisms of FA/CD36 regulation associated with obesity and/or type2 diabetes.

## Results and discussion

**Figure 1:**
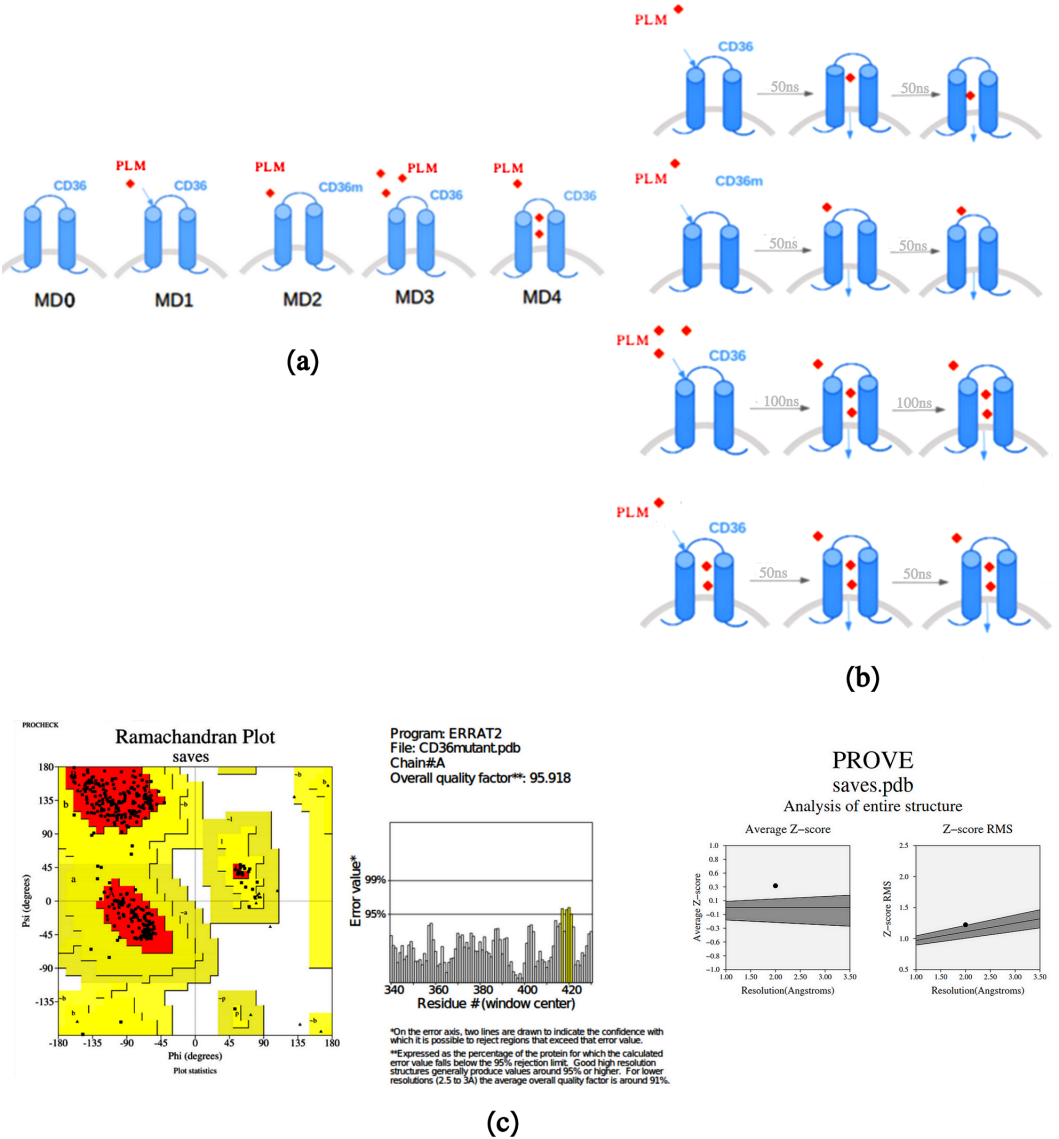
(**a**) Scheme of the molecular dynamics simulation procedure that the CD36 transporter (blue) and ligand plamitic acid PLM (reed). *MD0* The wild type CD36 protein was simulated in the absence of palmitic acid (PLM), *MD1* the wild type CD36 protein was simulated in the presence of a single PLM, *MD2* the mutant type CD36 protein was simulated in the presence of a single PLM, *MD3* the wild type protein was simulated in the presence of a three PLM put outside, *MD4* the wild type protein was simulated in the presence of a three PLM (two PLM put inside and one PLM outside). (**b**) Scheme of molecular dynamic results that the CD36 transporter (blue) and ligand plamitic acid PLM (reed) including the efflux of the substrate PLM along with the uptake across the CD36 receptor. (**c**) Evaluation of the mutant K164A CD36 (3D) structure by ERRAT2 and Prove and Ramachandran plot.

MD simulations can be used as a reply to many types of issues. Here we used MD simulations to determine the mechanism of transport Fatty acid (pathway and rate) CD36 receptor. Figure 1b illustrates an overview result of the applications of Molecular Dynamics Simulations, the timescale and the molecular motions accessible with the current simulation methods.

### Structural studies of mutational effects on fatty acid transport CD36 (K164A)

#### The three-dimensional (3D) structure of CD36 mutant (K164A)

The tridimensional structures of virtual mutant K164A ectodomain of CD36 predicted by using Swiss Model server to construct the homology model based on the crystal structure CD36-CIDRα (PDB ID: 5LGD). The quality of the homology model was evaluated by Ramachandran plot, the overall quality factor ERRAT2 plot and PROVE produced by the SAVS server v6.0 (https://saves.mbi.ucla.edu/). The stereo chemical properties of the modeled demonstrates that a majority of its residues are in favored region (92.2%) and allowed region (7.8%) of the Ramachandran plot, that indicates reasonable quality of this homology model. Moreover, the ERRAT2 score was 95.918 and the total quality index (Z-score) was 0.352. We observed that the currently mutant modeled structure have structural qualities that are acceptable^24^ (Fig. 1c).

#### Global molecular dynamics of the simulations analysis

Because protein flexibility represents a significant function in CD36 transport and signaling, we conducted MD simulations of 100 ns on wild type in absence of PLM, in addition to the Wild type and mutant type in the presence of PLM for further analysis. Also to study the link of Lys164 residues with PLM transport and binding, we first examined the interaction of CD36 wild type crystallized ecto-domain structure with (PDB Id: 5LGD) and models that were generated for the mutant CD36 in the presence of PLM as well as alone CD36 wild type using MD simulation. After 100 ps the structure of CD36w with PLM, revealed that PLM is located above Lys164 and near the upper helix. The room in the midst of group of carboxylic group of PLM and the Lys164 is 1.2 Å whereas the mutant residue Ala164 in helix I is 5.5 Å. Clearly, this shows that the mutant residue Ala164 is far from the binding site of CD36 (Fig. 1b) and it won’t instantly link with the PLM. Indeed, the mutation instantly impact the binding of PLM and hence, the K164A mutation has control of the overall conformation particularly, helix I, where the mutant amino acid K164A is existing. This is a pattern of the transportation channel of CD36 receptor^25^. CHO cells expressing mutated CD36 Lys-164 showed alteractions FA uptake and FA-induced calcium liberation indicating the importance of Lys-164 for both FA effects^20^.

**Figure 2:**
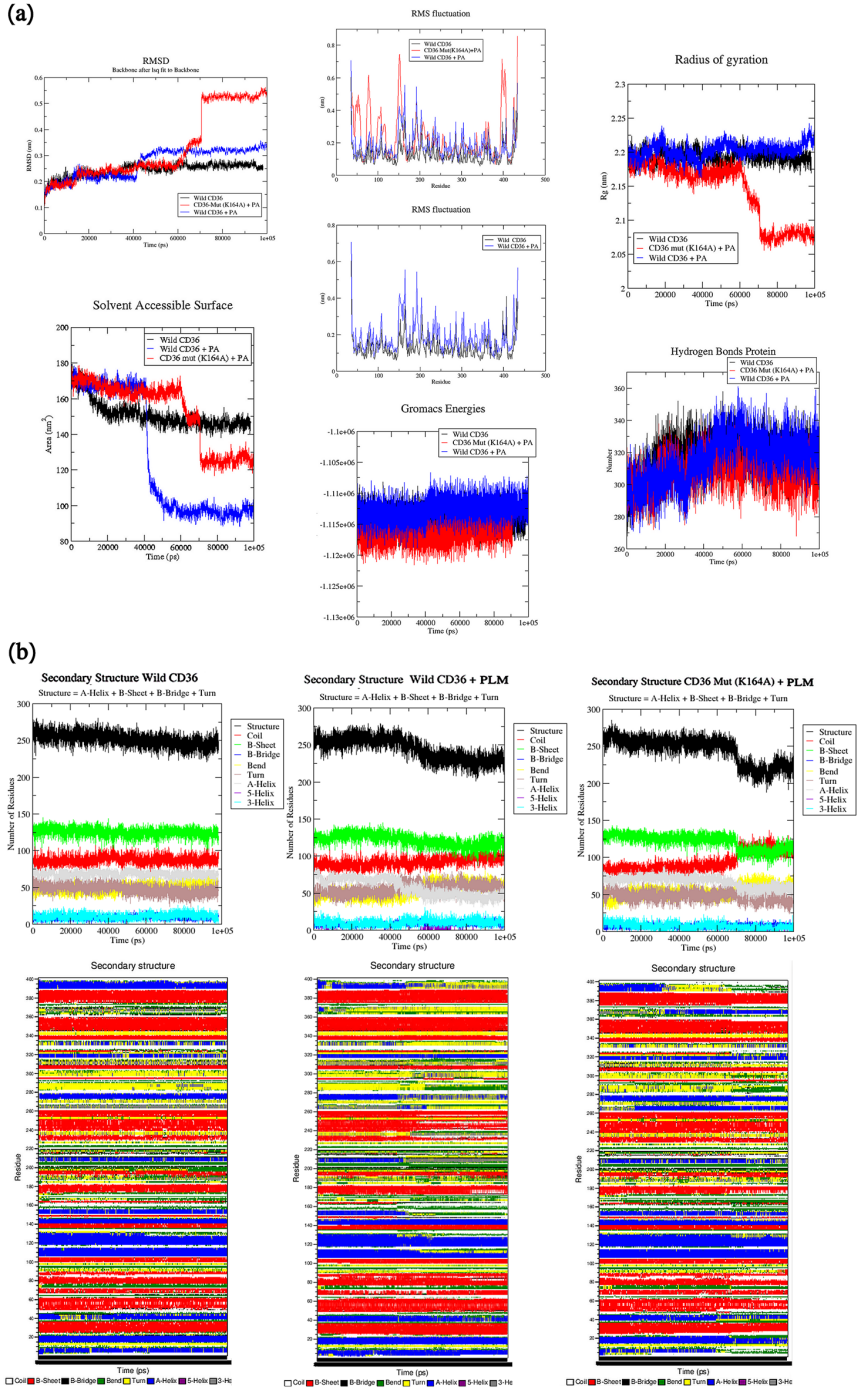
(**a**) Dynamics analyses of plamitic acid (PLM) uptake across the wild and mutant type CD36 receptor. RMSD plot, Backbone residual fluctuations (RMSF) plot, time evolution of Radius of Gyration (Rg), Solvent Accessible Surface (SASA), Gromacs Energies and Hydrogen Bonds for wild and mutant type of CD36 in presence and absence of PLM a function of time during 100,000 ps (100 ns). Black, blue, and red, represent values obtained for Wild type CD36 in absence of PLM, wild type CD36 in presence of a single PLM, mutant K164A type CD36 in e of in presence of a single PLM respectively. (**b**) DSSP analysis for the secondary structure fluctuations as a function of time from 0 to 100 ns of plamitic acid (PLM) uptake across the wild and mutant type CD36 receptor.

We calculated and evaluated the three simulation systems: the structure stability of the proteins in the time of the MD and RMSD/backbone, which were considered respective to the first conformation, as we had observed in the RMSD representation Fig. 2a. The plots of RMSD reflected equilibrium of the simulations that were signal of the global protein constancy. The peak RMSDs were at 0.25 nm to 0.55 nm for the three simulation systems. The plots were depicted in three sets. The first graph corresponded to CD36 wild-type only in absence of PLM, other showing the same for CD36 WT and MT in presence of PLM were illustrated separately with black, blue and red bars correspondingly. It is evident that the time taken for both the CD36-WT and CD36-MT in presence of PLM to reach convergence was more than their corresponding CD36r WT in absence PLM. The fluctuation size had a small difference in the medium RMSD value, which had a huge constancy from 38 ns. It was a dynamic constant comportment of the CD36r WT. The wild-type in presence of PLM attained early stability due to convergence from 43 ns whereas the mutated pair reached equilibrium much later.

The RMSF of C-alpha atoms was used to infer with the residue specific flexibility in order to study the fluctuation of every single residue. In other words, during MD simulation three RMSF plots for CD36 wild-type in absence of PLM, the CD36 wild-type and CD36m mutant in presence of PLM were generated Fig. 2a. For the bleu curve, a trimodal distribution at the regions close to the individual portal was observed for the complex CD36-PLM wild type compared to their wild-type only black curve. The elevation of RMSF at the upper helix domain and coil was supposed to be produced by the amino acid lys 164 impacts of first assays to enter from these portals, which surround the bottom of the cavity. Interestingly, CD36 mutant red curve, exhibited elevated flexibility at *N*-terminal region (from 50 to 160) and C-terminal at 400 end did not displayed a lot of displacement at the binding region Lys164. The difference in fluctuation patterns CD36 could be attributed to their subtle importance of Lys164 residues and structural features. The flexibility of the upper part of the helix/loop is important for the organization of the unlocking and locking of the path. The representation of the RMSF showed that the conformational order occurred in the mutant. The utmost modification has taken place in the loop and upper helix region. Clearly, the access of PLM from this portal requires protein conformational change in upper helix from Lys164, which might explain why it was the most preferred route in our multiple independent runs. To conclude the RMSF plot confirmed clearly that in the helix regions of the upper loop the fluctuations of the residues were more articulated. Since the radius of gyration (Rg) is a very powerful system in analyzing the configurational compactness, the form, and the coil of the global WT, WT-PLM and MT-PLM conformation at several period; we used it to executed Rg examination in order to look for the structural variations and dynamic stability of the wild type and mutate (K164A) structures in the presence of PLM. The average Rg values of WT, WT-PLM and MT-PLM from the trajectory were calculated as 2.2, 2.21 and 2.18 nm respectively see Fig. 2a. Throughout the simulation, the Wild type CD36 and complex wild type CD36-PLM are marked with black and blue lines respectively, exhibiting almost a similar pattern in terms of the Rg values on the contrary the complex Mutant Type CD36m-PLM which was marked with red line; This suggested that there was a change in the global conformation and coil of the preceptor after the mutation. The K164A mutation shows a significant decrease in Rg, implying a strong compactness that will influence the closure of the fatty acid transport cavity.

Moreover, in order to search for the hydrophobic center behavior of the wild type CD36 (WT) and mutant type (MT) structures in presence and absence of PLM, we had investigated the solvent surface accessibility area (SASA). The analysis of SASA displayed the residues of the complex MT-PLM structures CD36 (K634A) which had the same SASA estimation to the complex WT-PLM CD36 protein, and they both conserved the openability at 44 ns Fig. 1a and upper to WT in the absence of PLM. PLM made this amino acid more accessible to the solvent. An important decrease of the SASA mean was verified in the complex WT CD36-PLM. The CD36 K164A mutation which has brought about larger changes in the SASA value because it had a great surface area exposed to the solvent, this could cause an exposure of hydrophobic amino acids and subsequently the deployment of the receptor.

A calculation of hydrogen bond (HB) in CD36 WT, WT-PLM and MT-PLM mutants was given to interpret the stability and flexibility of the receptor. Figure 2a shows the quantity of intra molecular HB in the WT and mutant CD36 receptor in presence and absence of PLM throughout the simulation. The total of HB decreased in complex CD36 WT-PLM and MT-PLM mutants as compared to the WT at 40 ns. WT CD36 has 340 HB, whereas complex WT-PLM and MT-PLM have 320 HBs, respectively. This diminution in HB was considered a loss of conformation and compactness of the CD36 receptor in presence of PLM. Overall, a significant diminution MT-PLM after a simulation time of 40 ns disrupts the structure, dynamics, and the stability, which leads to a deregulated and dysfunctional CD36-PLM complex. Throughout the period of simulation, the energetic value of the complex WT-PLM is an indication of strong basis of the fact that the molecule has a stable structure as well as relatively higher amount of total interaction compared to complex MT-PLM. Destabilization in folding and thermodynamic stability may affect the total energy of the biomolecules. The results demonstrated a very slight rise in the total energy value in mutant structure this ruled out the notion of stability losses induced by mutation.

The secondary structural propensities become significant in exploring the structural properties of the protein. However, there were significant structural variations measured at each simulation time. These outcomes were consistent with the secondary structure evaluations with the DSSP utilizing the Fig. 2b. Therefore, this gave us a clear evidence that the secondary and tertiary constructing (a-helix and b-sheets) of the CD36 WT-PLM and MT-PLM structures were not stable during the simulation. The mutation K164A induced change in the A-helix content but it delayed at 70 ns on the contrary in WT-PLM at 40 ns. Our MD analysis has clearly showed that K164 change conformational, dynamics, and flexibility might be a possible cause of transport of fatty acid.

#### Principal component analysis PCA

**Figure 3:**
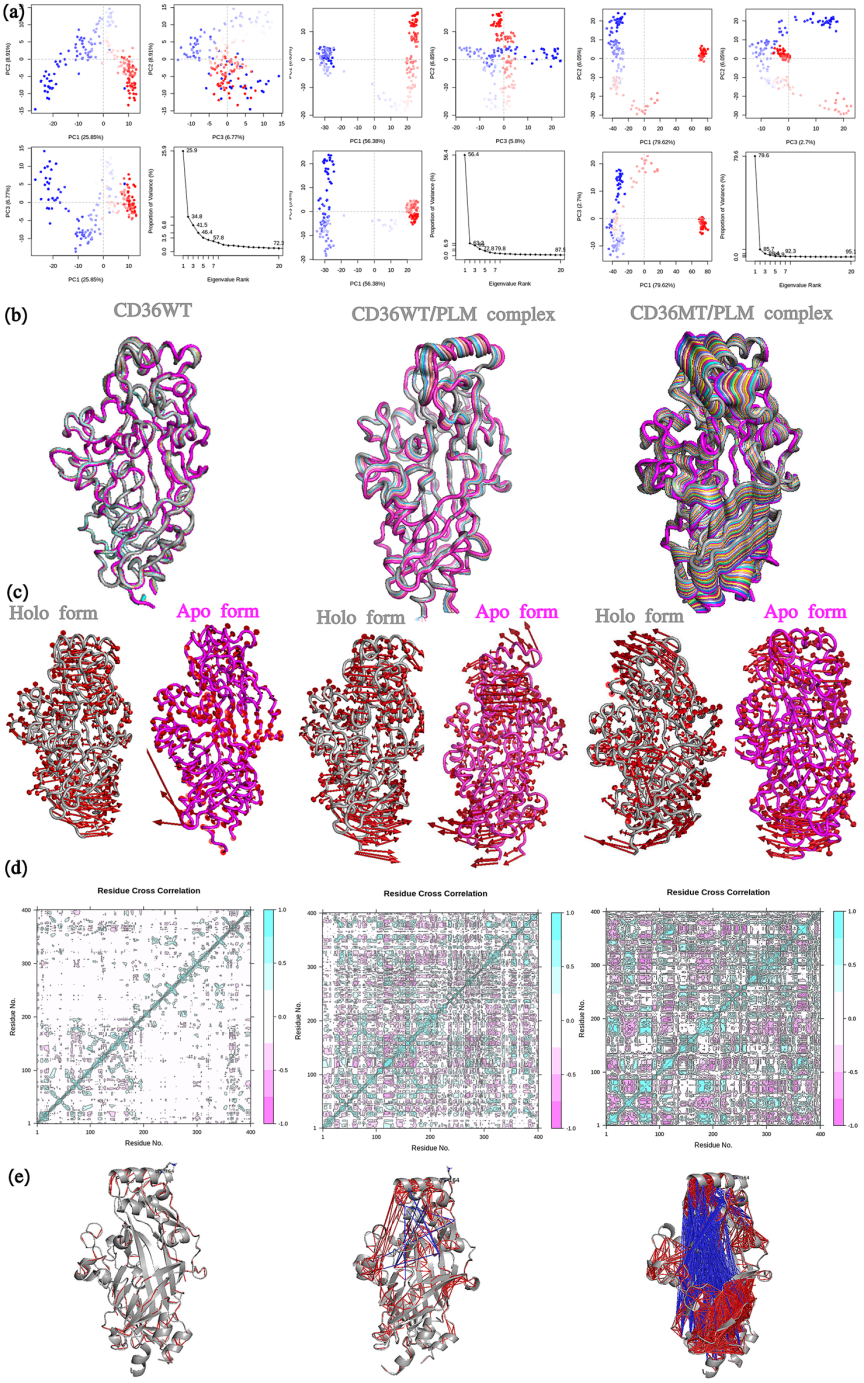
(**a–c**) Model and motion produced by principal component analysis for wild and mutant type of CD36 in presence and absence of PLM a function of time during 100,000 ps (100 ns). (**a**) Principal component analysis, PC2 vs PC1, PC2 vs PC3, PC3 vs PC1 and an eigenvalue rank plot. (**b**) Visualization of the motions along PC1. (**c**) Motion of two different modes by ANM Analyze, the red arrows show the direction and amplitudes of the movements occurred from first trajectory of holo and apo form in (gray) and (pink) color respectively. (**d,e**) Cross-correlation Analysis matrix of the fluctuations of the C α atoms of the residue from their average during 100 ns for wild and mutant type of CD36 in presence and absence of PLM. (**d**) Cross-correlation map for MD simulation. Cyan color indicates a positive correlation between residue fluctuations, while the pink color depicts a negative correlation. (**e**) Residue cross-correlations. CD36 is depicted by gray cartoon. Red and blue lines indicate correlated and anti-correlation motions.

We use the Principal component analysis (PCA) in order to identify the motions and the direction of the flexibilities in the residues. The principal components analysis was applied to the backbone atoms for CD36 WT, WT-PLM and MT-PLM such analysis showed that the first account 25%, 56% and 79% of variances of the total variance observed in the simulation data respectively Fig. 3a.

The first outstanding featured motions during the simulation were analyzed through secondary structures which includes an opening-closing motion of transport tunnel. Our observation focused on the movements of the C-coil, A-helix. We found, that the flexible motions of theses secondary structures in the distinct active states are quite dissimilar from CD36 wild type (WT) in absence of PLM and complexes CD36 WT-PLM and MT-PLM Fig. 3a–c. The top principal components for model PC1 for CD36 WT in absence PLM, WT-PLM and MT-PLM are illustrated in Fig. 3b. In the top two eigenvectors, the most prominent motions observed are propeller loops of CD36 MT-PLM which are the most flexible in moving in the opposite direction. Particularly, in holo structure the propeller loops attached to the upper, moves leftwards or rightwards for CD36WT and it moves in the opposite direction upwards to downwards for the CD36 MT in presence of PLM. In contrast, the helix and coil at termination K164 has an expanding or contracting movements in the WT in the presence of PLM, which is the opposite in the WT only in absence of PLM. Inclusively, the mutation results in a change in the movement patterns of these secondary elements. We have visualized this movement of the top two eigenvectors Fig. 3c: they show the orientation and the volume of selected eigenvectors by each of the backbone atoms. Although, there might be differences between the simulations with respect to the movement sampled, it is evident that this may suggest that the site K164 is able to settle the paradigm by reducing the movement of the loops to a reduce module.

#### Covariance analysis and motions

For a preferable comprehension of the association through the movement of the upper helix, coil and Lys164 residue, as well as the movements of the adjoining part, covariance analyses were executed during each of the separate simulation on the three groups CD36 WT, WT-PLM and MT-PLM. They were illustrated utilizing 3D system plots. As shown in Fig. 3d, positive correlations are mapped in light blue green color while negative correlations are mapped in pink color. The deeper color indicates more correlations (or anti-correlations).

We detected a cluster with correlated motions in the middle the secondary structures and the adjoining zone in the active situation of the related groups. The correlated motions of these parts grow extremely in the CD36MT-PLM state Fig. 3e correlation and anticorrelation resented in red and blue color line. However, the mutation K164A increased the anticorrelated motions between the upper helix and C-terminal and N terminal (tunnel). In addition, strongly correlated motions were also observed within the beta sheets regions and helix C-terminal and N-terminal regions, which signal that the conjunction of these parts retired and proposed that the flexibility of the helix coil is in charge for the movements of the adjoining pathways. Besides, the movements of these two secondary structures are correlated with the movement of upper helix that is essential for the structural stability of CD36 structure for PLM transport.

In the next parts, we revealed cavities in the several conformations during the trajectory for the six MD. A more functional study has indicate the elaborated organizational connection in the midst the motions of the upper helix, and coils, along with the movements of the adjoining conduits. The channels in holo conformations of CD36 crystal structure.

#### Transport channel analysis

**Figure 4:**
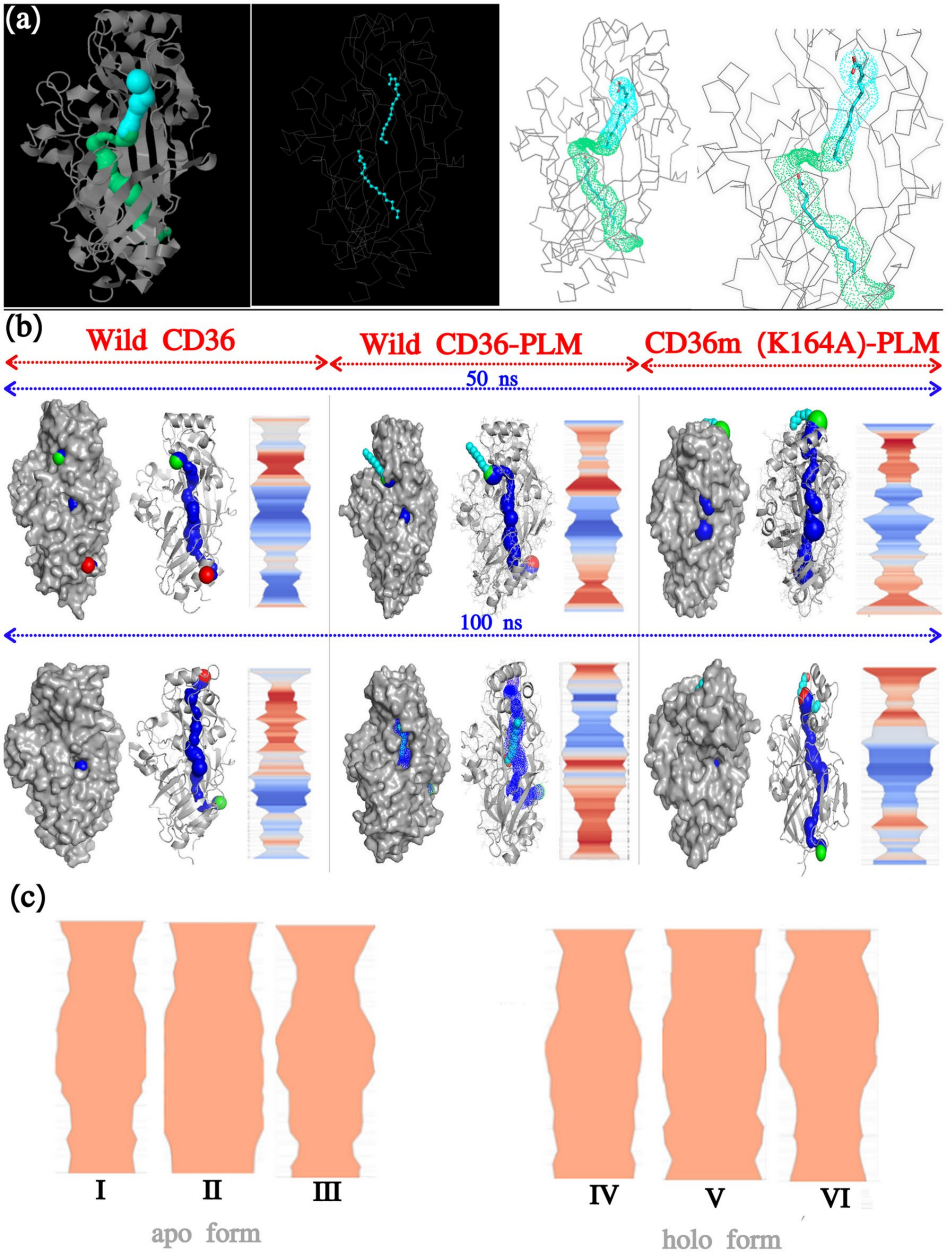
Structure of the studied CD36 with their respective tunnels. (**a**) PLM-bound snapshot, with the crystal structure of the wild-type CD36 (CD36-CIDRα, PDB-ID: 5LGD) depicting fully formed tunnel (identified using CAVER 3.0 PyMOL plugin). The main tunnel is shown by the blue and green surface. (**b**) CHEXVIS was used to analyze snapshots from 50 and 100 ns molecular dynamics simulation for six simulations for wild and mutant type of CD36 in presence and absence of PLM. (**c**) The transport channel of the fatty acid in the apo and holo conformations were identified based on the PCA analysis using CHEXVIS for CD36 WT, CD36WT–PLM and CD36MT–PLM.

The channels in holo conformations of CD36 crystal structure with two co-crystal of PLM^14^ were identified and analyzed using CAVER (Fig. 4a) in order to determine the associated motion between upper helix and the transport channel. To examine mutation effect K164A and active area of transport channel bottleneck amino acids were got by study the Channel Extraction and Visualization (CHEXVIS) during MD simulations this observed the conformation constituents Table 1.

**Table 1:** Cavity’s profile as seen by the ChExVis program.

Simulations	Rank	Pt 1	Pt 2	Length	Bottle-neck	Straightness	Score
Wild CD36 (holo form)	1	2	7	59.978	4.059	0.903	0.844
Wild CD36–PLM (holo form)	1	3	4	55.923	4.243	0.876	0.852
CD36m–PLM (holo form)	1	1	9	62.427	3.592	0.821	0.795
Wild CD36 (apo form)	1	3	10	48.392	3.746	0.858	0.826
Wild CD36–PLM (apo form)	1	4	6	59.430	4.264	0.895	0.835
CD36m–PA (apo form)	1	5	7	75.344	3.754	0.769	0.868

CHEXVIS was used to study Scenes from 100 ns MD for CD36WT, CD36WT-PLM and CD36MT-PLM; Fig. 4b represents tunnel width, length, and bottleneck width after 50 ns and 100 ns. The width indicats the extreme flexible domain for fatty acid, the bottleneck can elucidate the hardness of absorption of fatty acid from receptor hole. A reduce bottleneck showed in CD36MT-PLM complex signals the problem of the receptor in absorption and transporting the fatty acid. From the above results, it can be concluded that K164A influence helix conformation and the ability of the site closing and binding and the transport of PLM. The transport channel of the fatty acid in the apo and holo conformations were identified based on the PCA analysis using CHEXVIS for CD36WT, CD36WT-PLM and CD36MT-PLM Table 1 and Fig. 4c. The width channel profiles in 2D of holo structure has enlarged, that demonstrate the unlocking of the channel, as indicate in Fig. 4c,IV,V,I (The bottle-neck 4.059, 4.243, 3.592 and Length 59.978, 55.923, 62.427 for CD36 WT, WT-PLM and MT-PLM channel respectively) In contrast, the channel in the apo conformation showed a decline in the width of channel especially at bottle-neck level of, as shown in Fig. 4c,I,II,III).The bottle-neck 3.746, 4.264, 3.754 and Length 48.392, 59.430, 75.344 for WT, WT-PLM and MT-PLM channel respectively. In addition, only the width of the channel for CD36 MT-PLM of holo form was very small in bottle-neck Fig. 4c,VI remains in the inactive state correspond to an inaccessible pathway for substrate. It also it indicate that the mutation influences the conduit unlocking-locking by modifying the movement of the upper helix and loops. However, an issue appears related the way the mutation influences the movement of the helix and loops.

#### Analysis of properties of helix

**Figure 5:**
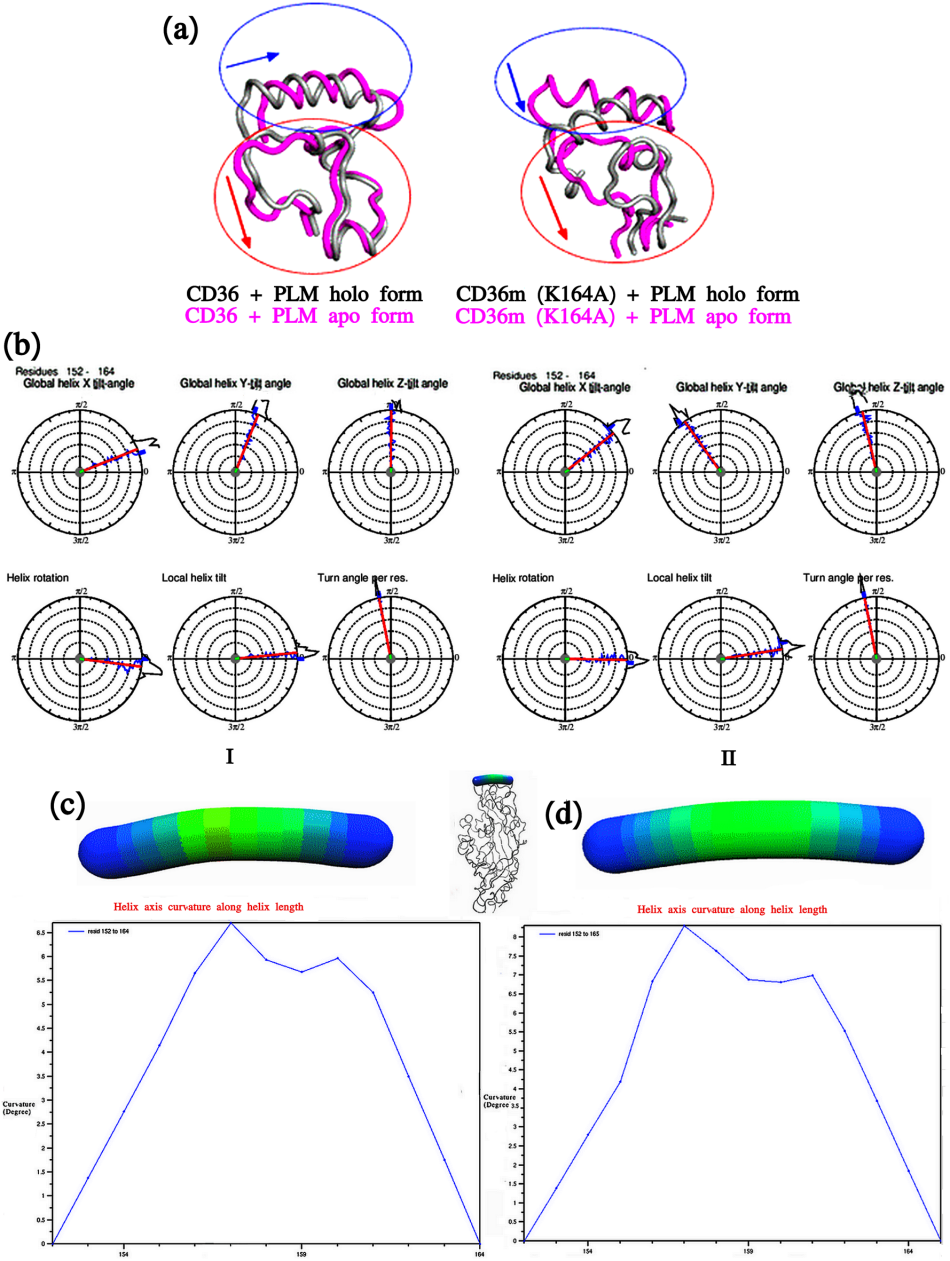
Properties of Protein helice during MD simulations. (**a**) Motion analysis based on the PCA analysis in apo (pink color) and holo (gray color) form of CD36 WT and MT in presence of PLM. (**b**) The geometrical parameters used for the analysis of the upper helix conformations. The values of the rotation angle calculated for the upper helices are plotted as a function of time for one of the MD simulations of CD36 WT (I) and MT (II) in presence of PLM. The angle is estimated with respect to the original inward facing structure. The blue ticks in the source and on the side of the circles measured the angle value for the first and last trajectory frames respectively; the red line corresponds to the mean angle value along the trajectory. (**c,d**) Analyze a static peptide’s local angles along the length of the helix and heatmap color represented a angle-indicative graphics of CD36 wild type (**c**) and CD36 mutant type (**d**) in presence of PLM.

To determine the associated motion between the Lys164 residue and C-coil and the surrounding channels, a precedent research proposed that the upper helix plays a function in stability between the unlocking and locking structures of CD36. The K164A mutation based on the PCA analysis, both the coil and the helix of the wild-types and mutants switch in contrary orientations in apo and holo form Fig. 5a. In order to provide the detailed view of the observed transitions, we have analyzed the MD trajectories for CD36 WT-PLM and MT-PLM complexes by calculating the average geometrical parameters of upper helix using Simulaid software. K164 residues, therefore, directly contribute to its global helix that was turned towards the right of Y axis Fig. 5b,I, while the mutation A164 mobility turned towards the left of Y and Z axis Fig. 5b,II. Lysine side chains, allowed the detection of their rotation in our simulations. As a result, it retains the helix rotation, local helix litl and turn helix per residue.

To study the mechanism of the rotation in our simulations of the mutation on the upper helix of CD36 proteins, we concentrated on the structure analysis of upper helix using Bendix analyses it analyzed static peptide’s local angles along the length of the helix showed in Fig. 5c,d. The result of the analyses of static peptide’s local angles along the length of the helix had 152–164 residue and the helical angles were measured with 6.5 curvature degree for WT-PLM Fig. 5c in comparison with MT-PLM Fig. 5d the length of the helix 152–165 residue. And the helical angles were measured with 8 curvature degree. On the whole, our investigation of upper helix proposes that the upper helix at the MT-PLM mutate situation of CD36 receptor tends to be wrapped, that influences the movement of the helix and loops of opening fatty acid transport site as revealed by the covariance analysis.

### Portals PLM transport and pathway

**Figure 6:**
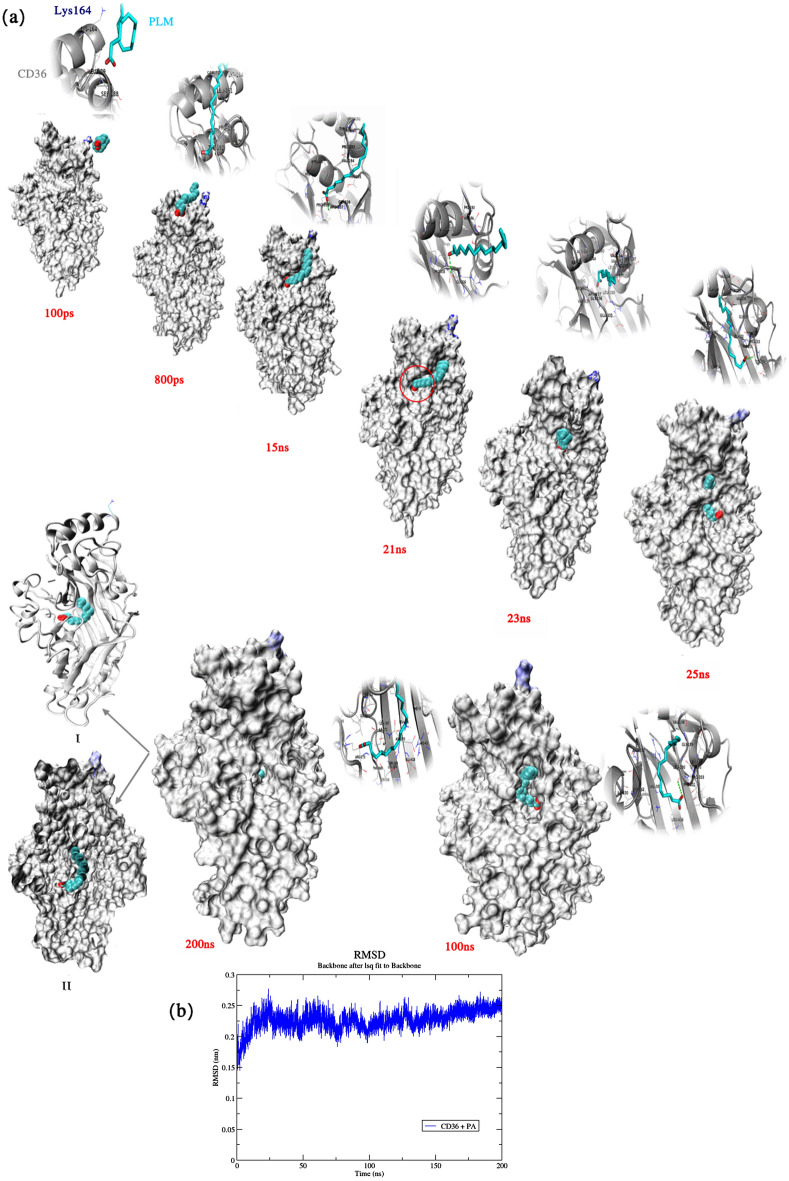
A snapshots representing the pathway of palmitic acid al transport at the portal of the CD36. (**a**) The snapshots correspond with 100 ps, 800 ps, 15 ns, 21 ns, 23 ns, 25 ns, 100 ns, 200 ns with one FA adsorbed to the protein. (**b**) The RMSD of the system, as they evolve with time. A is representation of Fatty acids at the starting point of the simulation 200 ns.

In the X-ray structure of CD36-complex, the first PLM occupied the channel connecting the helical portal region and the inner cavity thus^14^, if the helical portal is the only choice to enter the cavity, does it exist an alternative portal that might allow the direct exchange between PLM? But there was no direct evidence showing the feasibility of such a process. In order to evaluate the feasibility of the structural dynamics underlying this hypothesis was directly applied to only PLM in ligated protein complex. A successful penetration of PLM from principal portal was observed Fig. 6.

Figure 6 presents snapshots at 100 ps, 800 ps, 15 ns, 21 ns, 23 ns, 25 ns, 100 ns, 200 ns presenting the pathway of PLM transport at the CD36 taken during a simulation, where one palmitic acid (PLM) were randomly located in the simulation box together with the crystal CD36 (PDB code: 5LGD) and records the RMSD calculated for the wholes system as it evolves with time. At about 100 ps after the initiation of the simulation Fig. 6a the PLM hydrophobic tail joined the Lys164 residue first one colored blue, due to hydrophobic and the electrostatic interaction to attach to the protein. 800 ps later, the tight interaction between PLM and Lys164, stimulated the conformation change caused by partially penetrating from the portal region initially. Then, it tried to slide over a loop dissociated from the gap between a loop and a-helix Fig. 6a (red circle) by the carboxylic part of a PLM molecule in the inner cavity of the protein. 15–150 ns later the PLM had accessed the inner channel and finished its way ted till the termination of the simulation. During the 200 ns/time, the FA had approached the central inner cavity protein. Residues, which were commonly encountered by the Palmitic acid during expulsion, were important for the constitution of individual portals. On the starting protein structure of our MD simulations, the constituting residues, identifying these residues, that are highlighted Fig. 6a are Arg63, Phe65, leu140, Ala143, Ala144, Ile148, Leu161, Ser162, Lys164, Leu187, Leu188, Leu189, Pro191, TYR192, Pro193, Val194, Thr195, Val198, Thr197, Gly199, Leu200, Phe201, Pro203, Tyr204, Thr207, Ser268, Ser269, Asp270, Ile271, Arg273, Ile330, Lys334, Glu335, Gly336, Arg337, Pro338, Ser339, Val339, Lys385, Leu387, Val389, Leu343, Ile341, Thr369, Leu391, Leu416, Glu418. Small openings at the portal regions were closed in the starting structure, however, certain parts of portal (e.g. Arg337, Thr195, Val194 at loop and Ala144, Ile148 at upper helical) showing the potentiality of creating an opening at this region. Since the PLM molecule bind in to Arg337 was close to this portal, so it would be the most favorable route. This portal was the most recorded one in multiple repetitions of MD simulations; as a result this portal is believed to be the only choice for Arg337. We therefore conducted MD simulations of 200 ns on wild type in presence of one PLM as shown in the RMSD profile Fig. 6b. The plots of RMSD revealed to be stable during simulation. The result indicates that Lys164 residue which provides the structural basis of forming an opening at the region of the principal portal for the dissociation of palmitic acid molecules from this path.

### Fatty acid transport rates

**Figure 7:**
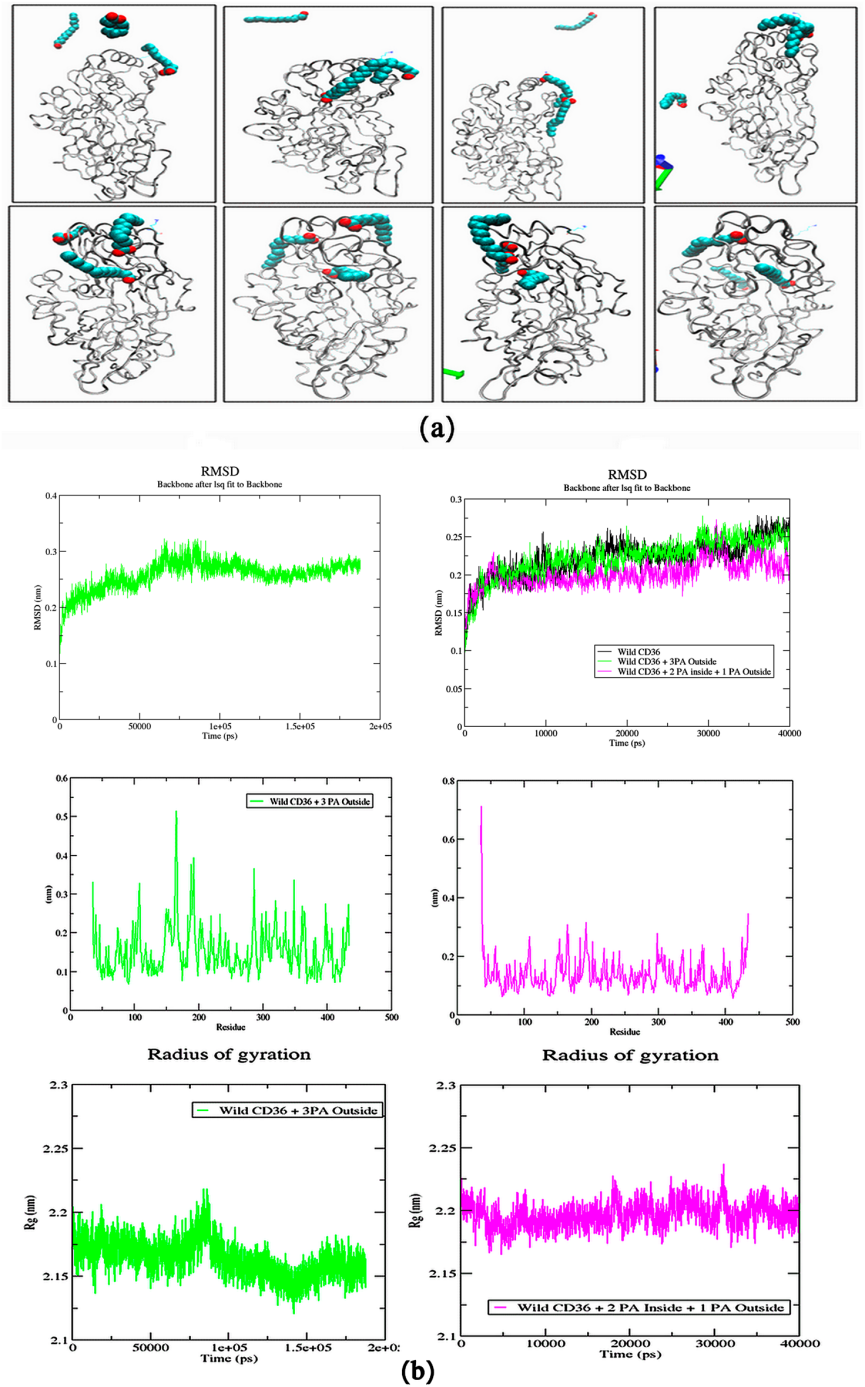
(**a**) Snapshots representing of Fatty acid transport rates, snapshots at 100 ps, 50, 100, 150, and 200 ns taken during each simulation, which depicted the pathway, transport mode and rate of three PLM to the portal CD36 receptor. (**b**) MD analyses of three PLM (three put outside two PLM put inside (green color) and one PLM outside (pink color)) Uptake across the wild type CD36 receptor. RMSD plot, Backbone residual fluctuations (RMSF) plot, time evolution of radius of gyration (Rg).

Here we described the outcome of MD simulations, using high concentrations of palmitate molecules. In the X-ray structure of CD36 complex, two PLM crystallized as described elsewhere occupied the channel connecting the portal area^14^. Consequently, there were no hypothetical proposals of how it would absorb from the receptor and even the maximal rates of palmitic uptake. To determine the rate of the fatty acid transported via the CD36 receptor and it existence other intermediate portals were entry portals using the same MD parameters with those for one PLM. Figure 7a presents snapshots at 100 ps, 50,100, 150, and 200 ns taken during each simulation, which depicted the pathway, transport mode and rate of three PLM to the portal CD36 receptor. In this simulation, 200 ns long, a crystal human CD36 (PDB code 5LGD) was equilibrated with three palmitic acid (PLM) that were placed in the simulation box at random locations outside. Figure 7b records the RMSD, RMSF, Rg calculated for the whole systems for three PLM outside and three PLM (two inside and one outside) as it evolves with time simulation. At the beginning we started with simulation (5 ns) Fig. 7a, the first PLM approximated to Lys164 after, a second PLM molecule continued and the third molecule moved away of the protein range electrostatic attraction of one of the carboxylates to the positive charge of the Lys164 amine side chain. 100 ns later, the first PLM moved towards the central portal zone due to hydrophobic reaction and second continued and one single PLM into the interior hole of the receptor. The third PLM had stayed in a liberate structure until the stop of the simulation. So, it formed an dimmer in conflict connected to the receptor. During this time, the liberate PLM had approached the receptor a shortly, but none of them led to the creation of a stable structure. To ascertain that the encounter between the protein and the two PLM was not accidental; we repeated the simulation using the toad crystal structure (PDB Id: 5LGD) and the pre-formed of two PLM inside and one outside molecules. We found out that the PLM outside was not absorbed till the simulation was terminated at 40 ns. As seen in Fig. 7 after the results of the dynamics of the system with three PLMs that the protein had transported two PLMs one by one to confirm these results we determined that the encounter between the protein and the two PLM was not accidental. Indeed, the simulation was repeated using the toad the same crystal structure and a pre-formed of two PLM inside and one outside the molecules. The PLM outside was dissociated till the simulation was terminated at 18 ns. Another system had to be carried out to put two PLMs inside and one outside after the simulation of the third PLM always remained outside and according to the RMSD, RMSF and radius of gyration (Rg) analysis it was always stable by contribution to the first simulation three PLM ext. The fluctuations of protein structure variation, in the time of the absorption from the three PLM, was observed in Fig. 7b (The green and pink curves respectively).

The principal structure fluctuation, as expected, occurred at the proximity of the specific portal. For the green line, the augmentation of RMSF at the upper helix region and coil was thought to be affected by the amino acids impacts of the starting trys to exit from this area. The domain with the growing RMSF, immediately participated by the ligand entrance from the gateway, K164 helix I loop, surrounding the bottom of the cavity. The outside absorption of the three PLM from this gateway had imposed a structure conformational modification contrariwise of the system two PLM one inside and one outside. Clearly, the two PLM are the most preferred to CD36 structure stabilization. Our CD36 structure model therefore supported a model in which fatty acids move through the cavity, identifying just one main possible entrance and favorable positions through which translocating ligands moved as they went towards the membrane proximal exit and the plasma membrane.

## Conclusion

In our research we have applied MD simulations to obtain a picture of fatty acid uptake rates model systems via CD36 at a molecular level. Models which are consisting of palmitic acid and CD36 receptor were investigated.

The aim of our study was to gain more understanding of the role of Lys164 residue in Fatty acid uptakes and to find out the mechanism of the functioning of the complex CD36/Fatty acids. From the different simulations done we have obtained a picture of the CD36 wild and mutant K164 in the presence and in the absence of fatty acid PLM. These simulations reveal both the conformational changes induced by Lys164 residue and the altered interactions induced by the mutagenesis of surface lysine, adversely affecting protein folding. All this was done in accordance with numerous investigations, where the results have shown that mutation K164A has decreased the productivity of the function of the protein transportation. Our MD analysis demonstrates the mode of global uptake Fatty acid transport via CD3636 receptor. The parameters which were calculated from the theoretical work are in good qualitative agreement with the experimental results. The results of this study have provided the confidence to extend our fatty acid uptake rate model for further MD studies.

## Computational methods

### Preparation of wild type and mutant CD36 protein for MD

The tridimensional structures of virtual mutant K164A ectodomain of CD36 was predicted by using SwissModel server to construct the homology model based on the crystal structure CD36-CIDRα (PDB ID: 5LGD) which is obtained from the Protein Data Bank, where all ligands and water molecules in the protein crystal structure were deleted^26,27^. The entire sequence of human CD36 was taken from the UniProt Database (http://www.uniprot.org) with accession number P16671. The quality of the homology model was evaluated by Ramachandran plot^28^, and its overall quality factor ERRAT2^29^ plot and PROVE produced by the SAVS server v6.0 (https://saves.mbi.ucla.edu/).

### Molecular dynamics fatty acid transport protocols

All procedures for MD simulations leaded out for 100, 200 ns using the GROMACS program, version 4.5.5, using the GROMOS56A1 force field^30,31^ were simulated under fully periodic boundary conditions. All electrostatic reactions were processed including Particle Mesh Ewald (PME). So every bonds between hydrogen molecules was recondition utilizing the LINCS algorithm^32^. Water organization was constrained using rigid SETTLE algorithm^33^ and non-bonded reactions, a cut-based is used, which allowed us to use an integration step of 2 fs. The ligand topology files, as needed in GROMACS, were generated from the PRODRG server^34^ which was an automated server for topology generation (https://www.sites.google.com/site/vanaaltenlab/). The complex was solubilized using SPC in water and balanced by the raise of K+ and Cl− ions in a cubic and dodecahedron box.

Five different simulations were carried (as summarized in Fig. 1a). In the three first simulations MD, the wild and mutant type protein was simulated in the presence and absence of a single palmitic acid, located near its surface. In the last two simulations, three palmitic acids were randomly put in the simulation cell and the last simulation (three PLM put outside) and (two PLM put inside and one PLM outside) in protein surface of wild type.

MD simulation of each complex was performed in five consecutive stages: (1) energy minimization, (2) NVT heating, (3) NPT equilibration, (4) NPT pre-production simulation, and (5) production simulation. Besides, the time step for the simulations was 2 fs. All MD calculations for period 100 ns and 200 ns. The RMSD, RMSF, Rg, and SASA calculated using GROMACS module. Furthermore, the evolution of the secondary structure was followed using the DSSP and VMD programs^35,36^. Visual depiction was taken out employing PyMOL and VMD.

### Principal component analysis and PCA cross correlation analysis

The principal component analysis (PCA) and cross-correlation were performed using the bio3D package in R and the anisotropic network model (ENM) was generated with PyANM plugin in PyMOL^37,38^. Once a sufficient MD production runs had been completed, the most important component analysis is used to analyze the results from the MD simulations. PCA became an efficient and an excellent tool for the global extract motions and the correlated structural dynamics across many biological systems.

### Channel profiles analysis of the wild and mutant K164A type CD36 receptor

CHEXVIS was used to analyze visual framework from 100 ns MD simulation of wild type (WT) and mutant K164A (MT) CD36 receptor in both the presence and absence of PLM, which revealed the structural details of the tunnel which are the width, length, and bottleneck distance of the tunnel^39^. We used CAVER 3.0 35 to provide detailed characteristics of crystal transport pathways^40^.

### Analysis of properties of helix

Analysis of the rotational movements of upper helice was performed with help of the TRAJELIX tool of the Simulaid package (https://mezeim01.u.hpc.mssm.edu/simulaid/) Earlier versions of Simulaid (IRIS GL GRAPHICS VERSION) and VMD Bendix Plugin, Version 1.1^41–43^.
